# An essential role of the mouse synapse-associated protein Syap1 in circuits for spontaneous motor activity and rotarod balance

**DOI:** 10.1242/bio.042366

**Published:** 2019-05-22

**Authors:** Cora R. von Collenberg, Dominique Schmitt, Thomas Rülicke, Michael Sendtner, Robert Blum, Erich Buchner

**Affiliations:** 1 Institute of Clinical Neurobiology, University Hospital Würzburg, Versbacher Str. 5, 97078 Würzburg, Germany; 2 Institute of Laboratory Animal Science, University of Veterinary Medicine Vienna, 1210 Vienna, Austria

**Keywords:** *Syap1* knockout, Motor behaviour, Associative learning, Fear conditioning, Object recognition

## Abstract

Synapse-associated protein 1 (Syap1) is the mammalian homologue of synapse-associated protein of 47 kDa (Sap47) in *Drosophila*. Genetic deletion of Sap47 leads to deficiencies in short-term plasticity and associative memory processing in flies. In mice, Syap1 is prominently expressed in the nervous system, but its function is still unclear. We have generated *Syap1* knockout mice and tested motor behaviour and memory. These mice are viable and fertile but display distinct deficiencies in motor behaviour. Locomotor activity specifically appears to be reduced in early phases when voluntary movement is initiated. On the rotarod, a more demanding motor test involving control by sensory feedback, Syap1-deficient mice dramatically fail to adapt to accelerated speed or to a change in rotation direction. Syap1 is highly expressed in cerebellar Purkinje cells and cerebellar nuclei. Thus, this distinct motor phenotype could be due to a so-far unknown function of Syap1 in cerebellar sensorimotor control. The observed motor defects are highly specific since other tests in the modified SHIRPA exam, as well as cognitive tasks like novel object recognition, Pavlovian fear conditioning, anxiety-like behaviour in open field dark-light transition and elevated plus maze do not appear to be affected in *Syap1* knockout mice.

## Abstract (precis)

**Summary:** Knockout of the *Syap1* gene in mice causes a distinct motor behaviour phenotype characterised by reduced initial locomotor activity and impaired rotarod performance.

## INTRODUCTION

Synapse-associated protein 1 (Syap1) is a member of the synapse associated BSD domain protein family (Doerks et al., 2002). It has been discovered by characterizing antigens using a library of monoclonal antibodies against *Drosophila* head homogenates (Hofbauer et al., 2009; Reichmuth et al., 1995). One of these antibodies binds to fly neuropil and in particular to presynaptic boutons of glutamatergic larval motoneurons. In head homogenates, the antibody detects a protein of 47 kDa, which was termed synapse-associated protein of 47 kDa (Sap47). Cloning and subsequent genetic deletion of the *Sap47* gene revealed that Sap47 is not required for viability. However, knockout larvae showed defects in short-term plasticity and olfactory associative learning and memory (Saumweber et al., 2011). The human gene encoding the synapse-associated protein 1 (*SYAP1*) is located within chromosomal band Xp22.2, a region associated with mental retardation, developmental delay and autism spectrum disorder (Prasad et al., 2012; Sismani et al., 2011).

The function of mammalian Syap1 is largely unclear. Syap1 has been shown to be important for adipocyte differentiation from murine embryonic stem cells by stimulating phosphorylation of Akt1 kinase (Yao et al., 2013). In cultured mouse motoneurons, however, no change in Akt phosphorylation after *Syap1* knockout or knockdown has been observed (Schmitt et al., 2016). Furthermore, size and body weight of *Syap1* knockout male mice (Y/−) are undistinguishable from wild-type littermates (Y/+) (Schmitt et al., 2016). Thus, the lack of Syap1 in mice apparently does not impair general metabolism or lipid storage *in vivo*.

The distribution of Syap1 immunoreactivity in the mouse brain has recently been reported (Schmitt et al., 2016). Syap1 is widely found throughout synaptic neuropil with high concentrations in regions rich in glutamatergic synapses. In addition, it has been detected in perinuclear structures in close proximity to the Golgi apparatus of subgroups of neurons, suggesting that it may be involved in a more general process of vesicular trafficking in addition to its putative role in synaptic transmission.

Here we describe the first behavioural analysis of *Syap1* knockout mice. Our data show that male mice hemizygous for a *Syap1* null allele (*Syap1^Y/−^*) exhibit a distinct motor phenotype in the open field (OF) test and on the rotarod during change of speed or direction. The main phenotype of the mutants might point to a specific so-far unknown function of Syap1 in cerebellar circuits for motor control. These findings could be of clinical relevance for patients with mutations in the *SYAP1* gene on chromosome Xp22.2.

## RESULTS

Observation of voluntary behaviour and movements of *Syap1* knockout animals in their cages revealed that knockout males, compared to wild-type littermates, show less exploratory activity and require longer to restore normal motor activity post handling. We therefore performed standardized behavioural phenotyping of these animals. We conducted a modified SHIRPA exam (summarized in Table 1) covering 20 measures of sensorimotor functions and reflexes (Hatcher et al., 2001; Rogers et al., 1997). Most aspects of the behaviour of *Syap1* knockout animals were inconspicuous in comparison to their wild-type littermates, indicating that lack of Syap1 does not cause generalized neurological defects. However, *Syap1^Y/−^* mice showed a specific deficit in locomotor activity, assessed by the number of squares crossed within a given time (Table 1).

**Table 1.**
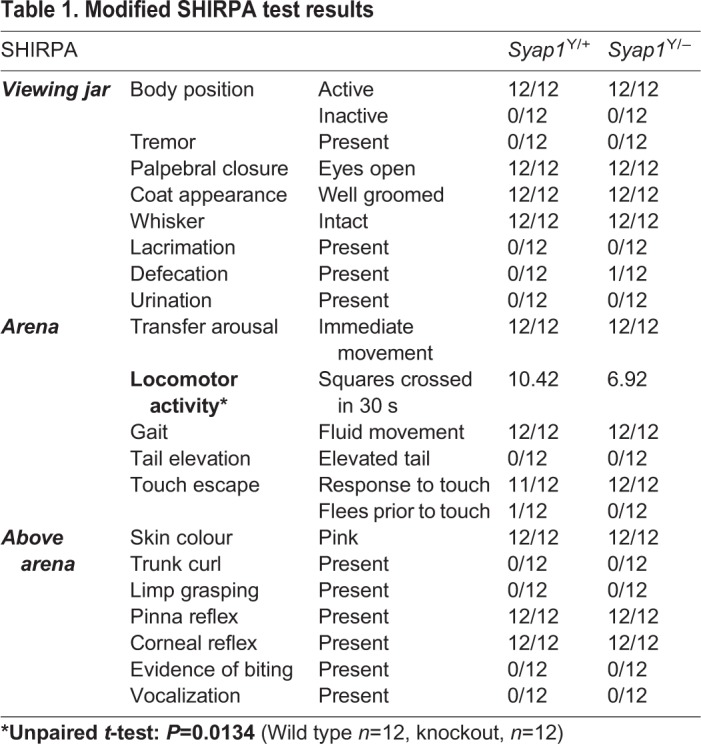
**Modified SHIRPA test results**

**Fig. 1.**
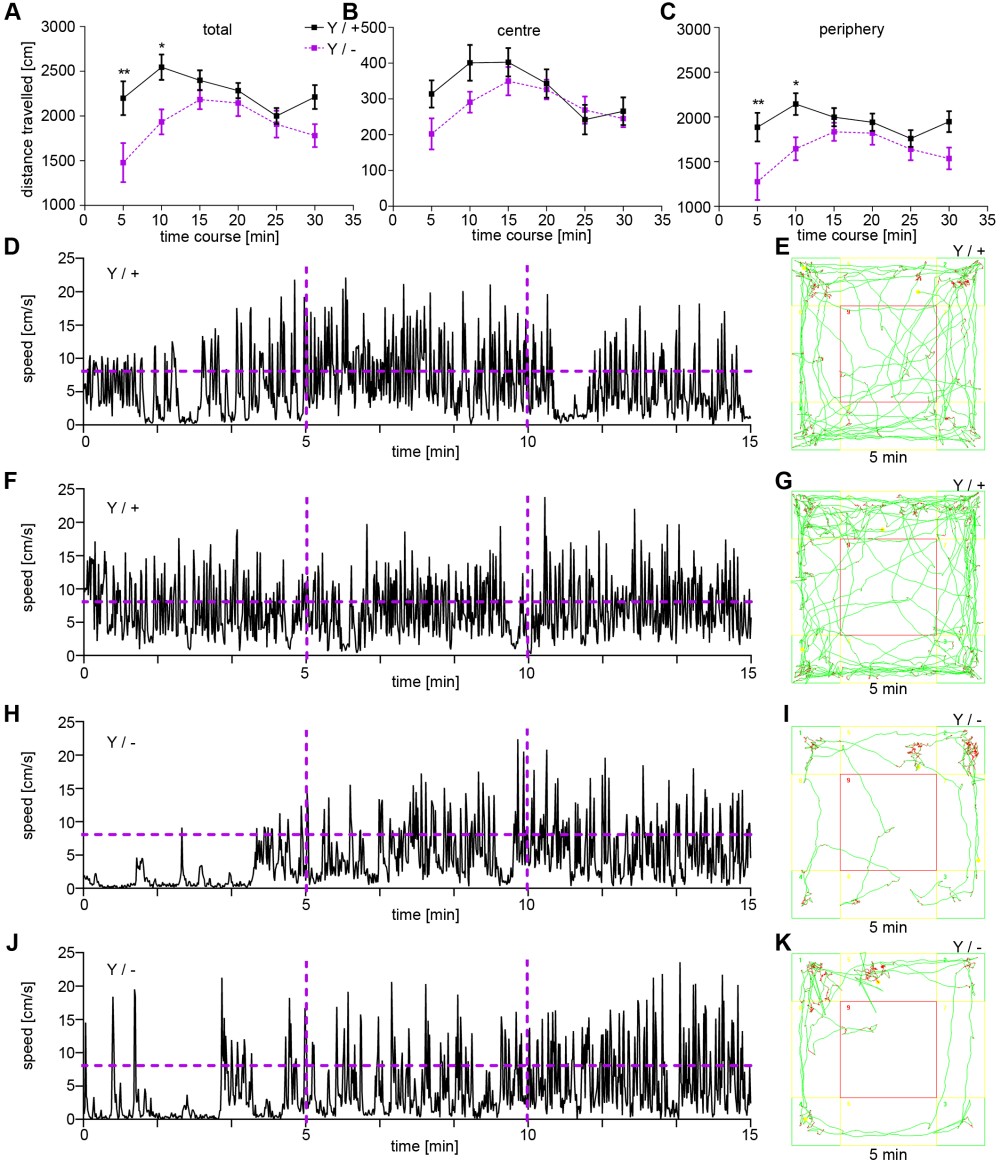
**Reduced locomotor activity of male *Syap1* knockout mice in early phases of the OF test.** (A) Total distance travelled per 5 min in the OF test. *Syap1* knockout animals travelled less within the first 10 min of the OF test (A, Sidak's multiple comparison: *P=0.0164, **P=0.0027, all other time points *P*>0.05). (B,C) Total distance travelled per 5 min periods in the centre and periphery of the OF test (Sidak's multiple comparison: *P=0.04, **P=0.0063). (D,F,H,J) Representative graphs showing the speed of movement for two wild-type and two *Syap1* knockout mice. The horizontal magenta lines indicate the mean overall speed of movement of wild-type mice (7.7 cm/s) and the vertical magenta lines separate time blocks of 5 min each. (E,G,I,K) Tracks showing the locomotor behaviour in the OF arena within the first 5 min of the test for wild-type (E,G) and *Syap1* knockout mice (I,K). Red indicates movement at a speed of less than 4.81 cm/s. Further statistical values are given in Table S1. (wild type *n*=12, knockout *n*=11).

The data acquired in the SHIRPA test point to a defect in motor behaviour. Therefore, we first performed the OF test to investigate the overall locomotor activity of *Syap1*-deficient mice (Carola et al., 2002; Hall, 1934). Notably, *Syap1* knockout mice showed a reduction in the total travel distances within the first 10 min in the OF test (Fig. 1A). This reduction was more pronounced in the periphery (Fig. 1C) than in the centre (Fig. 1B). *Syap1* knockout mice were more or less stationary during the first 5 min of the OF, before they regained their normal locomotor activity. Orientation-like movements on the same spot characterized these atypical early hypoactive phases. The atypical motor behaviour of *Syap1* knockout mice in the OF test is shown in Movies 1 and 2 (*Syap1^Y/−^*: Movie 1; wild type: Movie 2). This behaviour was clearly distinguishable from freezing behaviour, a typical defensive reaction of mice (Tovote et al., 2015). In the movement analysis, wild-type mice exhibited an overall mean speed of 7.7 cm/s, while *Syap1* knockout mice did not reach this mean speed (indicated as a magenta horizontal line in Fig. 1D,F,H,J). In later phases, after 15–30 min in the test arena, this phenotype was lost (Fig. 1A). We also plotted the locomotor speed over time to better illustrate this main effect (Fig. 1D–K). In later phases of the test, from min 15–30, *Syap1* knockout mice showed the capability to walk at similar speeds to wild-type littermates (Fig. 1D–K). Tracking for locomotor activity reveals that the *Syap1* knockout phenotype is mainly caused by the typical longer stationary phases (red labels in Fig. 1E,G,I,K). Significant differences in movement speed between the genotypes arose within the first 10 min of the OF test (Fig. S1A).

The OF test is also suited to examine anxiety-like behaviour in rodents (Carola et al., 2002; Hall, 1934). In contrast to the considerable changes in early locomotor activity, the time spent in the centre of the OF was not significantly different between the genotypes (Fig. S1B,C). The initial difference in the number of entries into centre and periphery (Fig. S1D,E) was most likely a consequence of the reduced initial locomotor activity.

**Fig. 2.**
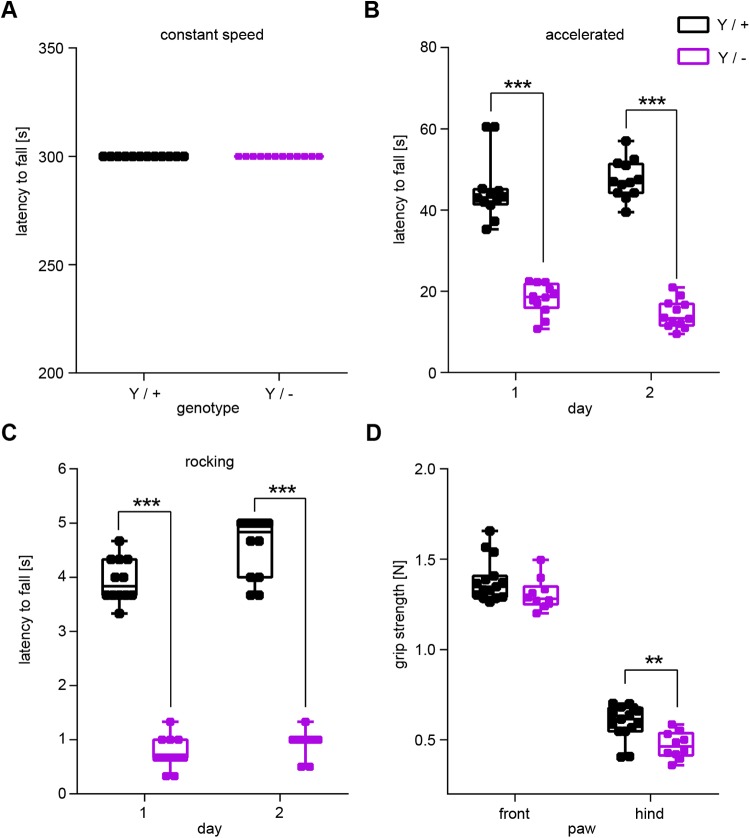
**Male *Syap1* knockout mice fail in the accelerated and rocking rotarod test.** (A) All animals managed to stay on a rod rotating at constant speed (5 rpm) for 5 min. (B,C) When rotation was accelerated (B) or reversed (C, rocking), latency to fall from the test apparatus was dramatically reduced in *Syap1* mutants (B,C, Sidak's multiple comparison: ***P<0.001, wild type *n*=12, knockout *n*=12). (D) Grip strength of the hind limb was slightly but significantly reduced in *Syap1* knockout mice (Sidak's multiple comparison: **P=0.0041, wild type *n*=15, knockout *n*=10). Further statistical values: Table S1.

A stringent test on motor coordination under proprioceptive feedback control is obtained when, instead of walking on a flat surface, the animal has to maintain its balance on a rotating rod (rotarod) (Jones and Roberts, 1968; Shiotsuki et al., 2010). Under the condition of steady rotation, *Syap1* knockout mice, like wild-type mice, managed to stay and walk on the rod for the entire observation period of 5 min (Fig. 2A). However, in more challenging situations, the *Syap1* mutants show clear deficiencies, like falling off the rod with very short latency when rod rotation was accelerated or reversed (rocking) (Fig. 2B,C). In both cases, the differences between the genotypes are highly significant (accelerated: *P*<0.0001; rocking: *P*<0.0001, two-way ANOVA). It is very unlikely that this effect was due to general muscle weakness, as grip strength was only slightly reduced in the hind limbs of the mutants (Fig. 2D, Sidak's multiple comparisons: *P*=0.0041). We also tested whether *Syap1^Y/−^* mice take advantage of voluntary running in a wheel. Notably, voluntary running distance overnight was not reduced in *Syap1* knockout mice compared to wild-type littermates (*Syap1^Y/−^*: 3.5 km; wild type: 3.6 km; mean of *n*=3 per genotype).

**Fig. 3.**
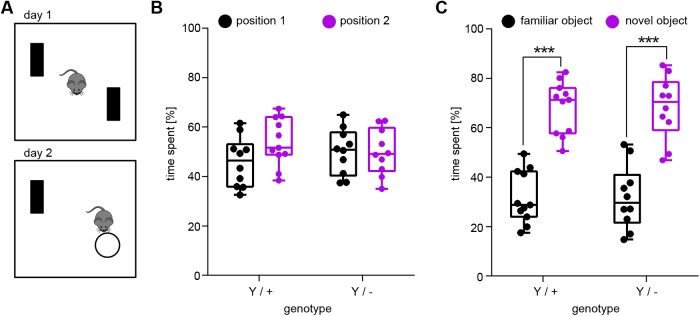
**Object recognition memory is not altered in *Syap1* knockout males.** (A) New object recognition paradigm. On the first day, the mice were confronted with two identical objects. 24 h later, the mice were placed back into the same arena, where one of the objects had been substituted with a novel one. (B) *Syap1* knockout and wild-type mice spent the same amount of time at either of two identical objects. Both genotypes spent significantly more time near the novel object on the next day (C, Sidak's multiple comparison: ***P<0.0001). The summary of the statistical values is given in Table S1. (wild type *n*=11, knockout *n*=10).

In *Drosophila*, knockout of the *Syap1* homologue *Sap47* causes impaired synaptic plasticity and reduced associative olfactory learning (Saumweber et al., 2011). We therefore investigated memory-related behaviour in *Syap1* knockout mice. We first performed the novel object recognition (NOR) test (Antunes and Biala, 2012; Ennaceur and Delacour, 1988). In the NOR test, the performance of the animals primarily relies on innate exploratory behaviour and does not involve reinforcement procedures such as food reward or electric foot shocks. In this task, a novel object needs to be noticed and processed as a memory trace. Furthermore, the pre-existing memory trace of the familiar object needs to be recalled after a certain delay (Ennaceur, 2010). Memory consolidation in the NOR test seems to be hippocampus-dependent and involves synaptic plasticity processes in the perirhinal cortex (Antunes and Biala, 2012; Rampon et al., 2000; Vignoli et al., 2016). The test was performed in the OF arena where mice were confronted with two identical objects on the first day (Fig. 3A). On the next day, one of the objects was substituted with a new one to see whether the mice remember the familiar object and spend more time exploring the novel object (Fig. 3A). Fig. 3B shows that *Syap1* knockout as well as the wild-type littermates spent almost equal amounts of time with two identical objects on day 1 (*P*=0.1907). On the next day, wild-type and *Syap1^Y/−^* mice both preferred the novel object (*P*<0.001), indicating that basic visual pattern recognition and pattern memory is not affected by Syap1 deficiency in mice (Fig. 3C). This shows that working memory and task-specific episodic memory elements are inconspicuous in *Syap1^Y/−^* mice.

**Fig. 4.**
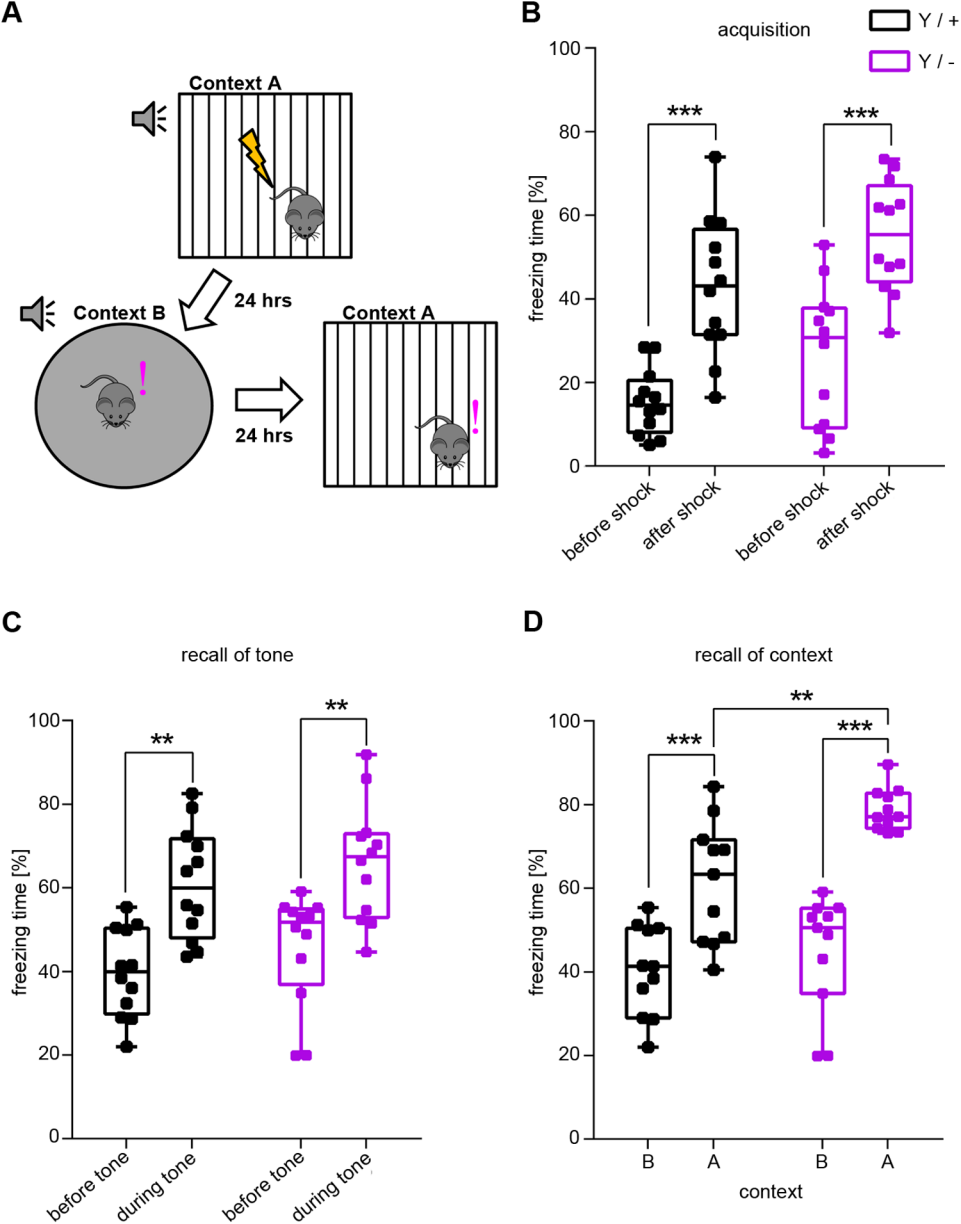
**Critical Pavlovian fear conditioning parameters are inconspicuous in *Syap1* knockout mice.** (A) Fear conditioning paradigm. Mice were conditioned in Context A, where an acoustic cue was followed by administration of an electric foot shock (unconditioned stimulus). One day later, mice were confronted with the same cue in a novel context (Context B). On the third day, mice were placed in Context A, but without cue presentation. (B) Fear acquisition. Wild-type and *Syap1* knockout mice displayed similar freezing behaviour after shock administration (Sidak's multiple comparison: ***P<0.001). (C) Tone recall in Context B. In Context B, both genotypes showed more freezing behaviour during tone presentation (Sidak's multiple comparison: ***P<0.001). (D) Context recall. Both genotypes freeze significantly more in Context A than in Context B (D, Sidak's multiple comparison: **P=0.0021, ***P<0.001). The summary of the statistical values is given in Table S1. (wild type *n*=12, knockout *n*=12).

We also tested *Syap1* knockout mice in a Pavlovian fear conditioning paradigm (Johansen et al., 2011; LeDoux, 2014; Tovote et al., 2015) that includes both cued and contextual fear conditioning (Fig. 4A). Pavlovian fear conditioning in mice is a typical paradigm to test for associative learning and memory processing (Johansen et al., 2011; LeDoux, 2014; Tovote et al., 2015). Plasticity defects in the amygdala are known to interfere with the conditioning of defensive behaviour to an auditory cue (tone) and to the training context, whereas conditioning to the training context (Context A), but not to the cue, is hippocampus-dependent (Maren and Hobin, 2007; Maren et al., 2013; Phillips and LeDoux, 1992). *Syap1* knockout mice displayed freezing behaviour similar to their wild-type littermates in both tests (Fig. 4). The freezing rate increased in both genotypes after initial shock administration indicating inconspicuous fear memory acquisition (Fig. 4B). A significant difference between genotypes (*P*=0.0194) was further revealed by two-way ANOVA. This genotype effect may however be caused by a difference in immobility prior to foot shock application (unpaired *t*-test, *P*=0.049). On the next day, when mice were placed in a new context (Context B) but were confronted with the same tone that preceded shock delivery the previous day (recall of tone), both knockout and wild-type mice spent significantly more time freezing during tone presentation (Fig. 4C). No significant genotype effect was detected in this case. On the third day, mice were again exposed to the training Context A. Compared to exposure to the neutral Context B on the previous day, the recall of the training context resulted in much higher amount of time spent freezing during Context A exposure (Fig. 4D). Here again, a genotype effect was detected which is reflected by longer duration of freezing of the *Syap1* knockout mice in Context A (Fig. 4D, Sidak's multiple comparison, *P*=0.0021). These data suggest that *Syap1^Y/−^* mice are able to process associative memory in the fear circuit, as indicated by the cue- and context-dependent elements of the conditioning paradigm. The data also show the ability for context discrimination in *Syap1^Y/−^* mice, a function attributed to the hippocampus (Frankland et al., 1998; Maren et al., 2013).

**Fig. 5.**
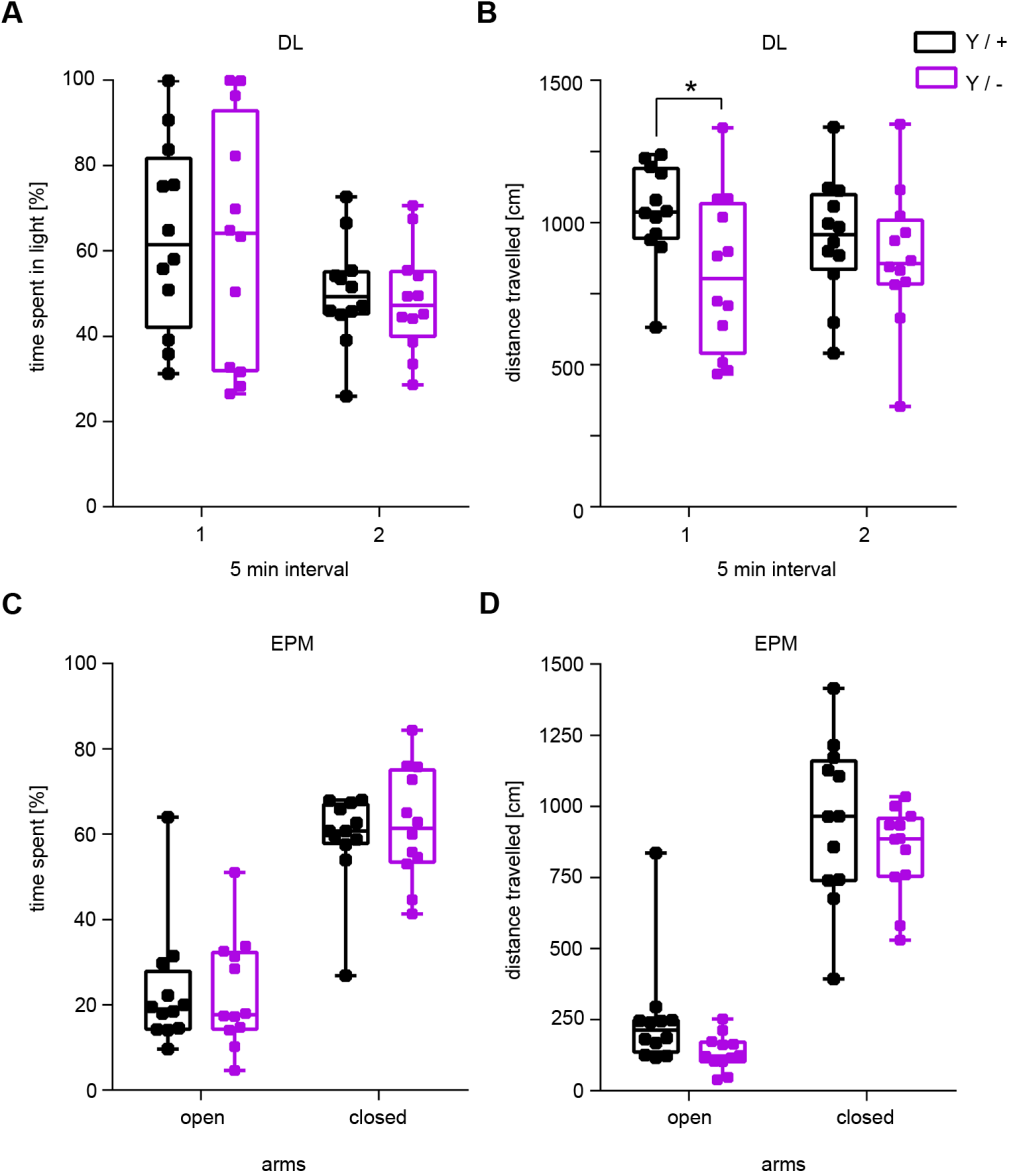
**No anxiety-like behaviour in male *Syap1* knockout mice in the dark-light transition and elevated plus maze tests.** (A) *Syap1* knockout mice and wild-type littermates spent an equivalent amount of time in the light and dark areas of the arena, indicating no anxiety-like behaviour in this test. (B) Within the first 5 min of the test, the distance travelled in the dark-light arena was slightly reduced in the mutant (Sidak's multiple comparison: *P=0.0471). (C) *Syap1* knockout mice and wild-type littermates spent the same amount of time on the open arm of the elevated plus maze. (D) In the elevated plus maze test, total distance travelled did not differ between the two genotypes. The summary of the statistical values is given in Table S1. (wild type *n*=12, knockout *n*=12).

Next, we performed two more tests to investigate explorative activity and anxiety-like behaviour; the dark-light (DL) transition test (Crawley and Goodwin, 1980) and the elevated plus-maze (EPM) test (Carola et al., 2002; Pellow et al., 1985). In a dark-light arena, no differences in the time spent in the illuminated light chamber versus the dark chamber were observed (Fig. 5A). However, as already seen in the SHIRPA and OF tests, reduced locomotor activity was indicated by fewer distances travelled by the *Syap1* mutants (Fig. 5B). In the EPM, independent of the genotype, mice spent more time in the closed arms than in the open arms of the test apparatus (Fig. 5C). Here, the distance travelled was inconspicuous between the genotypes (Fig. 5D). The DL transition test and the EPM are in accordance with the results obtained in the OF arena (Fig. 1, Fig. S1) and confirm that the reduced motor performance of *Syap1^Y/−^* mice is unlikely to be caused by increased anxiety-like behaviour.

**Fig. 6.**
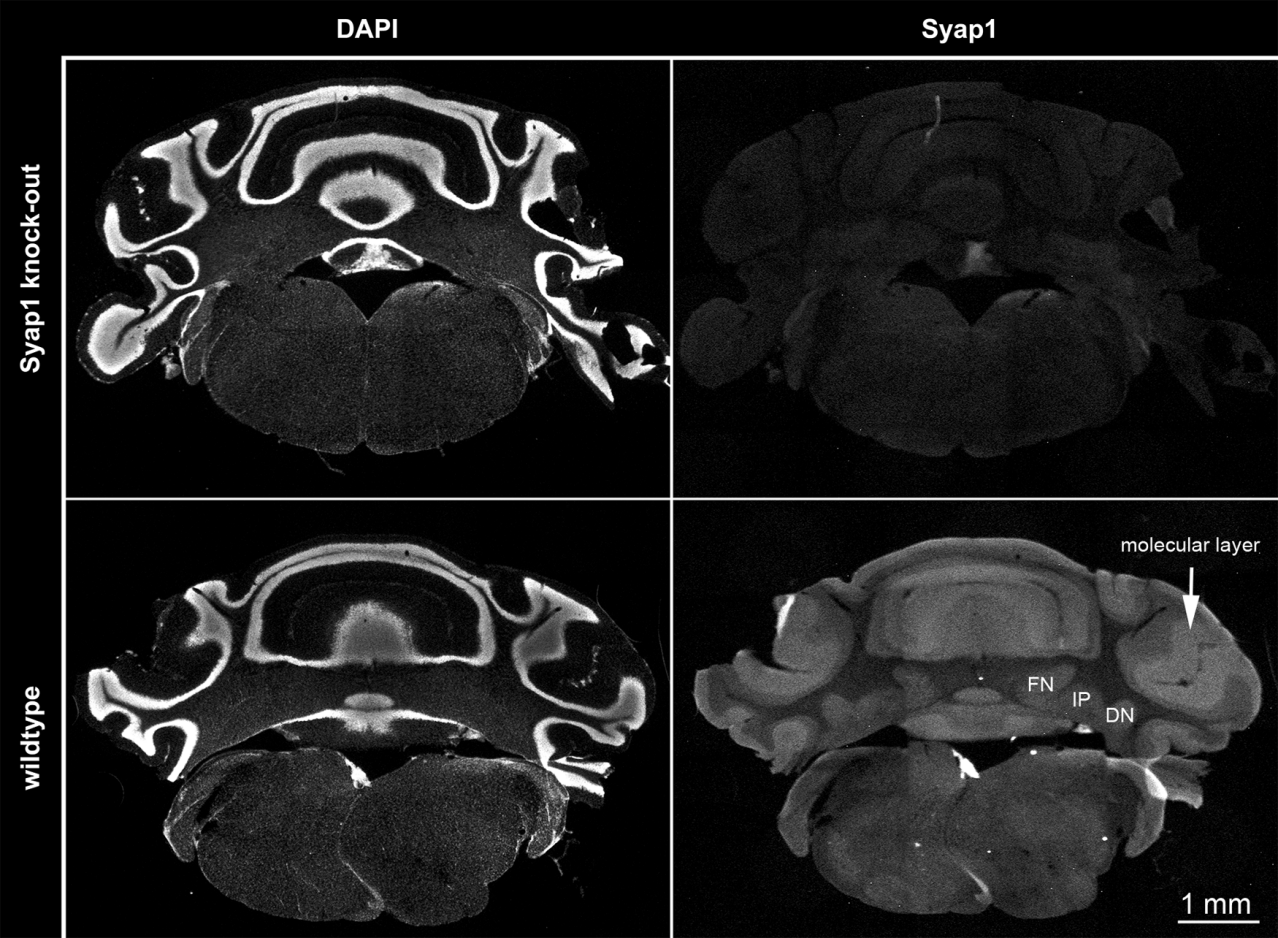
**Anti-Syap1 immunoreactivity in the cerebellum.** The images in the right panels show the immunofluorescence labelling of Syap1 protein in *Syap1* knockout (upper panels) and wild-type (lower panels) coronal sections of the cerebellum. DAPI labelling (left panels) was used as counterstain. Immunolabelling and image acquisition was performed as described earlier by Schmitt et al. (2016). The image illustrates the broad distribution of Syap1 protein in the cerebellum. High-resolution confocal analysis of the distribution of Syap1 in the cerebellar cortex has already been described earlier (Schmitt et al., 2016). These overview microscopy images illustrate the abundance of Syap1 in the deep cerebellar nuclei [fastigial nucleus (FN), interposed nucleus (IP), and dentate nucleus (DN)].

In a recent study we showed that Syap1 is highly abundant in the cerebellum (Schmitt et al., 2016), particularly in Purkinje cell somata and in the molecular layer. Notably, the protein is also highly expressed in the cerebellar nuclei (Fig. 6), indicating that Syap1 deficiency might affect the motor behaviour at different sites in the functional centres of the cerebellar motor control system.

## DISCUSSION

Syap1-deficient mice show a distinct locomotor phenotype, whereas in cognition-related behaviour tests they appear inconspicuous. The motor phenotype is most obvious in early phases of the OF and in the accelerated rotarod tests. Interestingly, mice spent more time being stationary in early phases of the OF test, when voluntary initiation of locomotion is needed. Notably, in the late phase of the OF test, Syap1-defienct mice show normal exploratory behaviour, indicating that the mice are able to adapt to the task. *Syap1^Y/−^* mice easily manage the rotating rod at a constant speed and take advantage of voluntary wheel running, but they fail in the accelerated or reversed rotarod. The data argue against a fundamental defect in reflexive, rhythmic, and voluntary motor performance or muscle contraction coordination, but point to distinct changes in subcortical motor control centres (Bostan and Strick, 2018; Calabresi et al., 2014; De Zeeuw and Ten Brinke, 2015; Grillner et al., 2005; Kiehn, 2016).

The motor control system in mammals is organized hierarchically, involving both cortical and subcortical circuits. In wild-type mice, Syap1 is strongly expressed in the cerebellum (Schmitt et al., 2016) and in cerebellar nuclei, particularly in Purkinje cell somata and in the molecular layer, where numerous glutamatergic synapses are formed between Purkinje cells and parallel and climbing fibres (Schmitt et al., 2016). The specific motor deficit in *Syap1* mutant mice may reflect changes in complex cerebellar motor control functions (De Zeeuw and Ten Brinke, 2015; Thach, 2014). Purkinje cells are the only efferent output from the cerebellar cortex and exert their function through projections to the deep cerebellar nuclei. Among the cerebellar nuclei, the fastigial nucleus (FN) is the phylogenetically oldest nucleus and is involved in axial, proximal and ocular motor control (Manto et al., 2012; Zhang et al., 2016). The FN is considered to be one of the ultimate output systems for cerebellar function and modulates motor behaviour via the vestibulospinal and reticulospinal tracts (Zhang et al., 2016). Defects in this projection pathway may explain the loss of function in the accelerated and reversed rotarod. Little is known about the role of the FN in the mouse, but studies in other mammals suggest that the FN also contributes to movement initiation via a bi-synaptic projection from the FN to the primary motor cortex (Kelly and Strick, 2003). This might contribute to the atypical motor behaviour in early phases of the OF test. Based on our results and considering the overall phenotype, we propose that cerebellar functions for fast adaptation to more complex movements and regulated motor initiation might be affected by deletion of Syap1 in mice. This would also be in accordance with the observation of a slightly reduced hind-limb performance in the grip-strength test.

In wild-type mice, Syap1 is also found in cortical areas involved in movement control (Schmitt et al., 2016). Therefore, we cannot exclude the possibility that the distinct motor behaviour in *Syap1* mutants is due to reduced capabilities to initiate motor behaviour or to a reduced internal motivation to explore the OF arena. Both functions are controlled by neural circuits that include dorsal or ventral striatal information processing (Bostan and Strick, 2018; Grillner et al., 2005). Our data support the view that Syap1-deficent mice are motivated and able to perform almost normally in less demanding movement tasks. The distinct motor phenotype observed in *Syap1^Y/−^* mice seems to be different from deficiencies observed in genetic or induced mouse models of Parkinson's disease, where subtle alterations in the nigrostriatal dopamine system are not accompanied by obvious impairments on the rotating rod or OF (Chesselet and Richter, 2011; Fleming et al., 2013; Lam et al., 2011; Lu et al., 2009; Sedelis et al., 2001). Furthermore, the normalization of the voluntary movement behaviour at later time points in the OF is suggestive of a specific motor initiation problem that is distinct from overall hypoactivity or anxiety-like behaviour typically observed in genetic mouse models for Parkinson's disease (Chesselet and Richter, 2011; Goldberg et al., 2003; Lam et al., 2011; Taylor et al., 2010). The *Syap1* mutants do not show a typical bradykinesia-like behaviour (Chesselet and Richter, 2011; Taylor et al., 2010) and no hyperactivity (Chesselet and Richter, 2011; Lam et al., 2011; Taylor et al., 2010). Parkinson patients often have substantial difficulties in starting locomotion. However, severe stages of disease also show bradykinesia or even more severe motor disabilities (Burn et al., 2006; Selikhova et al., 2009) that are generally accompanied by non-motor syndromes (Blum and Lesch, 2015; Rutten et al., 2015; Selikhova et al., 2009). However, anxiety-like behaviour and cognitive information processing in the NOR task and Pavlovian fear conditioning tasks appear largely normal in *Syap1^Y/−^* mice. Thus, Syap1-deficiency does not fundamentally disturb information processing in the corresponding cortical areas, the hippocampus or the amygdala.

In *Drosophila*, deletion of the *Sap47* gene causes a ∼50% decrease in larval learning scores for odour–tastant association (Saumweber et al., 2011), but motor performance of adult mutant flies is yet to be analysed. The lack of obvious defects of *Syap1* knockout mice in associative fear conditioning and novel object recognition, accompanied by a distinct motor skill deficit, does not necessarily point to a different molecular and cellular function of the evolutionarily conserved Sap47/Syap1 proteins, but might reflect a similar protein function expressed in the context of fundamentally different neural circuits.

In two human patients with autism spectrum disorder, copy number variants affecting the *SYAP1* gene have been observed (Prasad et al., 2012). At present, it is not clear whether SYAP1 dysfunction in humans causes any phenotype. The motor defects observed in the *Syap1* knockout mouse could be relevant for the discovery of such an association.

## CONCLUSION

We conclude that advanced motor skills, but not basic motor performance, depend on Syap1. Since it has been shown that Syap1 is highly concentrated in synaptic regions of cerebellar Purkinje cells (Schmitt et al., 2016), we suggest that there may be a causative link between cerebellar Syap1 expression and intact sensorimotor control. Basal ganglia and the cerebellum are interconnected at the subcortical level (Bostan and Strick, 2018). Therefore, we assume that the cerebellum and the dorsal striatum might represent target structures for a detailed analysis, which could lead to a better understanding of the molecular and cellular function of Syap1.

## MATERIALS AND METHODS

### Animals

All animal experiments were carried out in accordance with European regulations on animal experimentation and protocols were approved by the local authorities (licence number: 55.2.2-2532.2-558). Mice were housed individually and kept at a 12-h dark-light cycle with access to food and water *ad libitum*. The cages (Tecniplast, 1264C Eurostandard Typ II, 267×207×140 mm) were kept in a Scantainer (Scanbur Ltd, Denmark) assuring stable conditions by maintaining a temperature of about 21°C and air humidity of about 55% through a constant air flow. Generation and verification of the *Syap1* null allele has been described before (Schmitt et al., 2016). Knockout males (Y/−) and wild-type littermates (Y/+) aged between 11 and 19 weeks were used for the experiments which were carried out during the light phase. The experimenter was blinded regarding the genotype of the animals.

### Modified SHIRPA

Mice were individually placed in a viewing jar, a hollow acrylic glass cylinder of 18.7 cm height and 14.2 cm diameter, to observe and note down the following features: body position, tremor, palpebral closure, coat appearance, whisker appearance, lacrimation, defecation and urination. The next day, mice were placed in an arena (Tecniplast, 1284L Eurostandard Typ II L, 365×207×140 mm) and the following aspects were observed: transfer arousal, locomotor activity, gait, tail elevation and touch escape. The following features were also evaluated: skin colour, trunk curl, limp grasping, pinna reflex, corneal reflex, evidence of biting and vocalization.

### Open field

A white square box made of frosted plastic (48×48 cm, height 50 cm), evenly illuminated with ca. 40 lux was used as an OF arena. Mice were placed in the middle of the arena and were monitored for 30 min using a web cam-based system (camera: Logitech). Animal movements were tracked and analysed with Video Mot Software (TSE, Germany). For analysis, the floor of the box was divided into different fields of interest. The following parameters were measured and compared between the centre of the arena (24×24 cm) versus the periphery: total distance travelled over time, travelling speed, time spent in the centre or periphery of the arena, and number of entries to the centre and into the periphery.

### Rotarod

Motor skills were also analysed on the rotarod (Ugo Basile). In the first test phase, mice were investigated at a continuous speed of 5 rpm for 5 min. In the second test phase, the rotarod was set to accelerate from 5 rpm to ∼50 rpm within 5 min. In the third test phase (reverse rocking), mice were placed on the rod, which was programmed to alternate rotating forwards and backwards to a final speed of 5 rpm. Accelerated and rocking rotarod were each performed on two subsequent days. For all test phases, the latency to fall off the rod was measured in seconds.

### Grip strength

Grip strength of front and hind paws was measured as the obtained tension peak in Newton with a digital force gauge (Chattilon Digital Force DFI2). The procedure was repeated four times to acquire a mean value of grip strength.

### Novel object recognition (NOR)

The novel object recognition test was carried out in the OF arena (Leger et al., 2013). Two objects were presented: a cell culture flask (T75 Greiner, height about 16 cm) filled with water to the top and a 14.6 cm tower built of Lego bricks. Objects were placed in two diagonally opposing corners of the box 12 cm from each wall. During the first day, two identical objects were presented in a randomized fashion. Mice were observed and tracked for 10 min using the Video Mot Software (TSE Germany, camera: Logitech). The time the mice spent with their head in the fields of interest (2 cm distance surrounding each object) was measured manually. On the second day, mice were confronted with a familiar and a novel object. Here, a triplicate of the objects was used to avoid olfactory cues or influence. Position of objects was randomized to avoid position bias.

### Pavlovian fear conditioning

For Pavlovian fear conditioning, the Multi Conditioning System from TSE (256060 series) was used. The animals were monitored and tracked with the TSE MCS FCS–SQ MED software. On the first day, mice were placed in Context A, a square clear acrylic box placed on a metal grid. After a habituation time of 60 s a tone was presented three times (CS, intensity 85 dB SPL, 10,000 Hz) that lasted 10 s with an inter stimulus interval (ISI) of 20 s. The CS was accompanied by a foot shock of 0.7 mA (US) which was delivered via the electric grid during the last second of tone presentation. After an additional time of 30 s, mice were transferred back to their home cage. To test for cue memory the next day, mice were placed in Context B, a clear acrylic cylinder placed on black, rough plastic. After an initial time of 60 s, the CS (tone) was delivered again three times for 10 s with an ISI of 20 s, without administration of the US. On the third day, mice were placed back in the fear conditioning context (training Context A) for 6 min without CS presentation to recall contextual memory.

### Dark-light transition

The dark-light transition test was performed in the OF arena. For this purpose, a red acrylic glass box of 47×16×25 cm was positioned in the box covering approximately one third of the arena with a square opening serving as an entrance for the mouse to get into the dark compartment. Mice were placed in the light compartment and their movements were tracked for 10 min using the Video Mot Software (TSE Germany, camera: Logitech). The following parameters were recorded and analysed: distance travelled in the light compartment and time spent in each compartment.

### Elevated plus maze

The elevated plus maze consisted of a cross with two closed and two open arms made of white frosted plastic (TSE Germany; length of arm: 30 cm, width: 5 cm, height of closed arm: 15 cm, height above ground: 48 cm; luminosity adjusted to ca. 60 lux). Mice were placed on one of the open arms and their movement was tracked for 10 min using the Video Mot Software (TSE Germany, camera: Logitech). The following parameters were analysed and compared between open and closed arms: distance travelled and time spent in open and closed arms.

### Statistical analysis

All statistical analyses were performed with GraphPad Prism 6. Data are presented as means with standard error of the means (±s.e.m.). To account for statistical differences between genotypes and conditions depending on the experiment, two-way ANOVAs with *post-hoc* Sidak's multiple comparison tests or unpaired *t*-tests were performed. Results were considered statistically significant at *P*<0.05. A summary of the statistical results is given in Table S1.

### Immunofluorescence labelling and microscopy

Immunofluorescence labelling and microscopy were performed using the affinity-purified rabbit anti-human Syap1 antibody (cat# 16272-1-AP; Proteintech, Chicago, USA, dilution: 1/200), as described in detail previously (Schmitt et al., 2016).

## Supplementary Material

Supplementary information
