# Correction: An essential role of the mouse synapse-associated protein Syap1 in circuits for spontaneous motor activity and rotarod balance

**DOI:** 10.1242/bio.048942

**Published:** 2020-02-18

**Authors:** Cora R. von Collenberg, Dominique Schmitt, Thomas Rü licke, Michael Sendtner, Robert Blum, Erich Buchner

There were errors in Biology Open (2019) 8, bio042366 (doi:10.1242/bio.042366).

The wrong *y*-axis label was included in Fig. 2C. The corrected and original figure are shown below and both the online full-text and PDF versions of the article have been updated.

**Fig. 2C (corrected panel).**
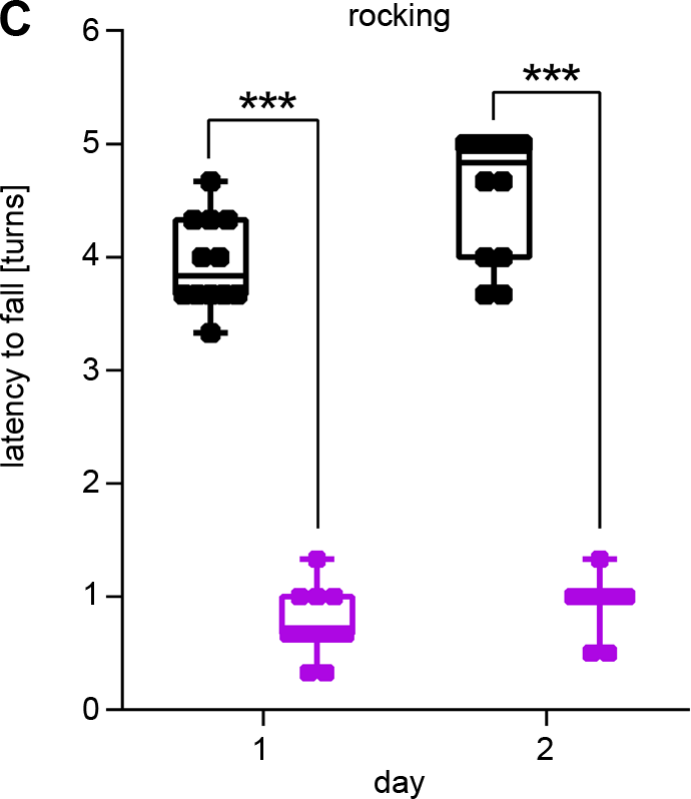

**Fig. 2C (original panel).**
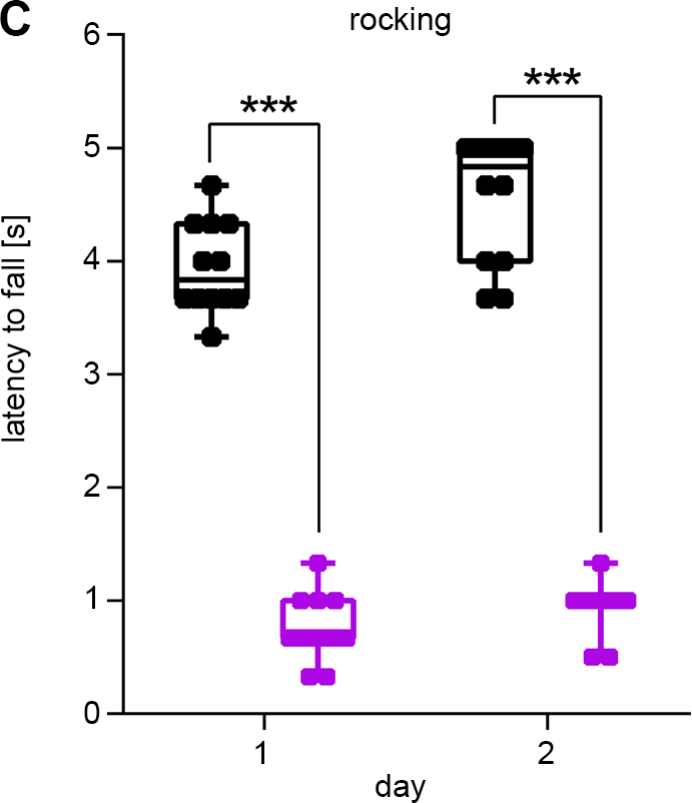

The wrong information was inadvertently included in the ‘Rotarod’ paragraph in the Materials and Methods section. The corrected and original text are shown below and both the online full-text and PDF versions of the article have been updated.

**Rotarod (corrected text)**

Motor skills were also analysed on the rotarod (Ugo Basile). In a first test phase, mice were investigated at a continuous speed of 20 rpm for 5 min. In a second test phase, the rotarod was set to accelerate from 15 rpm to ∼50 rpm within 20 sec. In a third test phase (reverse rocking), mice were placed on the rod programmed to alternate rotating forwards and backwards starting at a speed of 15 rpm to a final speed of 40 rpm within 20 sec followed by a change in direction. This cycle of accelerated speed followed by a change in direction was repeated five times. Accelerated and rocking rotarod were each performed on two subsequent days. For all test phases, the latency to fall off the rod was measured in seconds or turns.

**Rotarod (original text)**

Motor skills were also analysed on the rotarod (Ugo Basile). In the first test phase, mice were investigated at a continuous speed of 5 rpm for 5 min. In the second test phase, the rotarod was set to accelerate from 5 rpm to ∼50 rpm within 5 min. In the third test phase (reverse rocking), mice were placed on the rod, which was programmed to alternate rotating forwards and backwards to a final speed of 5 rpm. Accelerated and rocking rotarod were each performed on two subsequent days. For all test phases, the latency to fall off the rod was measured in seconds.

The authors apologise for these errors and any inconvenience they may have caused.

This correction does not affect the results in the article or the conclusions of this study.

