# Supplementary material for: An essential role of the mouse synapse-associated protein Syap1 in circuits for spontaneous motor activity and rotarod balance
Source: Biol Open. 2019 May 22;8(6):bio042366. doi: 10.1242/bio.042366 (PMC6602322; doi:10.1242/bio.042366)
Supplement: Supplementary information [file biolopen-8-042366-s1.pdf]

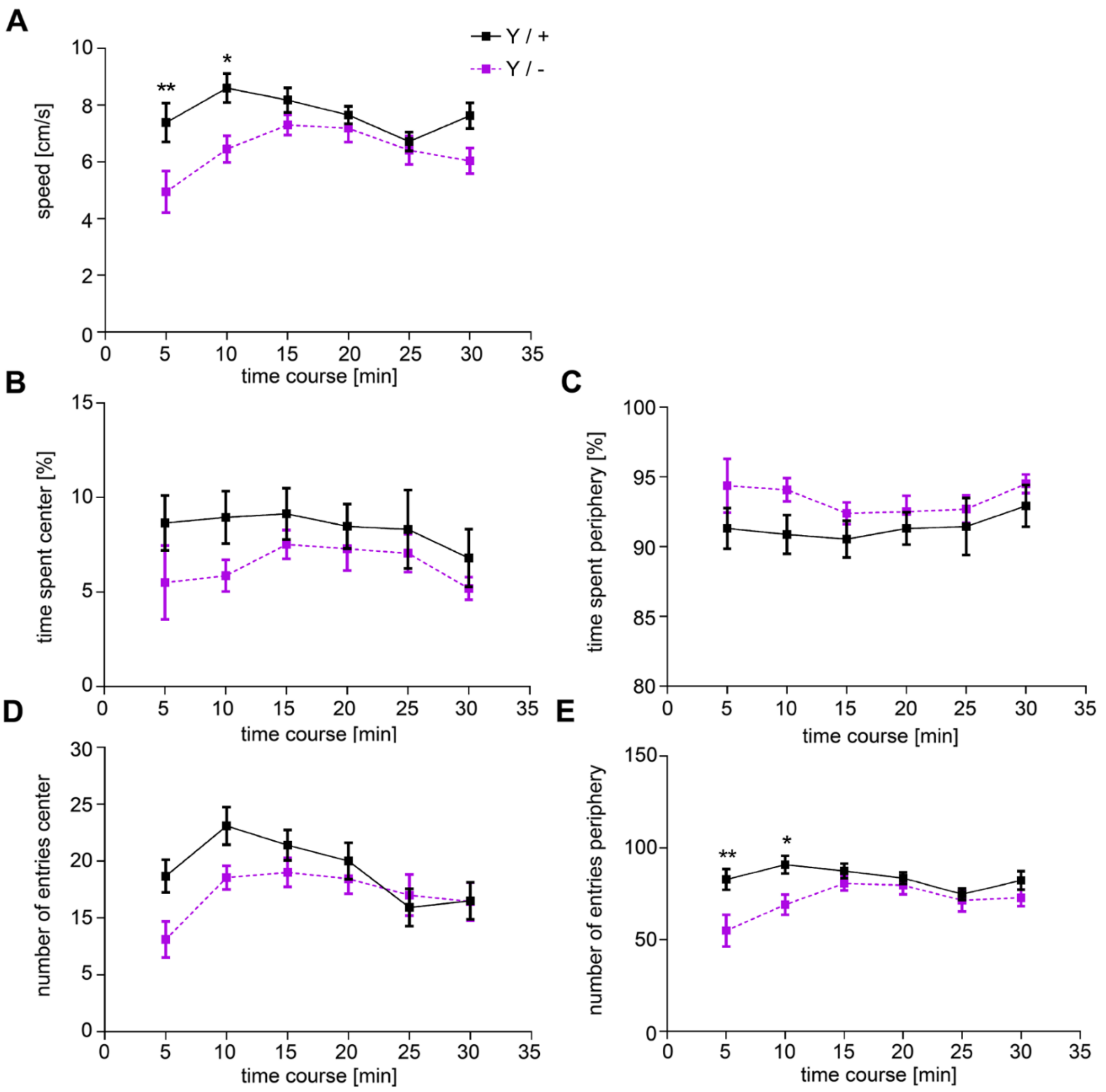

Figure S1. Open field test parameters.

(A) Speed of *Syap1* knockout and wildtype mice over time (Sidak's multiple comparison: * p = 0.0150, ** p = 0.0040). (B, C) Time spent in the centre or periphery of the open field arena. (D, E) Number of entries into the centre or periphery of the open field arena (C, Sidak's multiple comparison: * p = 0.0197, ** p = 0.0011). The summary of the statistical values is given in supplementary table 1. (wildtype n = 12, knockout n = 11)

Table S1: Statistical Analysis, two-way ANOVA

**Fig. 1 A**					
ANOVA table	SS	DF	MS	F (DFn, DFd)	P value
Interaction	1,951e+006	5	390232	F (5, 105) = 2,191	P = 0,0607
Time course	3,879e+006	5	775703	F (5, 105) = 4,356	P = 0,0012
Genotype	4,665e+006	1	4,665e+006	F (1, 21) = 9,642	P = 0,0054
**Fig. 1 B**					
ANOVA table	SS	DF	MS	F (DFn, DFd)	P value
Interaction	87812	5	17562	F (5, 105) = 1,303	P = 0,2683
Time course	339521	5	67904	F (5, 105) = 5,038	P = 0,0004
Genotype	77454	1	77454	F (1, 21) = 2,253	P = 0,1482
**Fig. 1 C**					
ANOVA table	SS	DF	MS	F (DFn, DFd)	P value
Interaction	1,317e+006	5	263492	F (5, 105) = 1,896	P = 0,1013
Time course	2,040e+006	5	408094	F (5, 105) = 2,936	P = 0,0160
Genotype	3,541e+006	1	3,541e+006	F (1, 21) = 8,029	P = 0,0100
**Fig. 2 B**					
ANOVA table	SS	DF	MS	F (DFn, DFd)	P value
Interaction	121,4	1	121,4	F (1, 22) = 8,651	P = 0,0076
Day	4,278	1	4,278	F (1, 22) = 0,3049	P = 0,5864
Genotype	10780	1	10780	F (1, 22) = 262,6	P < 0,0001
**Fig. 2 C**					
ANOVA table	SS	DF	MS	F (DFn, DFd)	P value
Interaction	0,5250	1	0,5250	F (1, 22) = 3,847	P = 0,0626
Day	1,944	1	1,944	F (1, 22) = 14,25	P = 0,0010
Genotype	139,0	1	139,0	F (1, 22) = 846,1	P < 0,0001
**Fig. 2 D**					
ANOVA table	SS	DF	MS	F (DFn, DFd)	P value
Interaction	0,008471	1	0,008471	F (1, 46) = 0,9126	P = 0,3444
Paw	7,867	1	7,867	F (1, 46) = 847,5	P < 0,0001
Genotype	0,1245	1	0,1245	F (1, 46) = 13,42	P = 0,0006
**Fig. 3 B**					
ANOVA table	SS	DF	MS	F (DFn, DFd)	P value
Interaction	171,9	1	171,9	F (1, 36) = 1,822	P = 0,1855
Genotype	4,547e-013	1	4,547e-013	F (1, 36) = 4,821e-015	P > 0,9999
Position	167,8	1	167,8	F (1, 36) = 1,779	P = 0,1907
**Fig. 3 C**					
ANOVA table	SS	DF	MS	F (DFn, DFd)	P value
Interaction	2,604	1	2,604	F (1, 36) = 0,01807	P = 0,8938
Genotype	-1,819e-012	1	-1,819e-012	F (1, 36) = -1,263e-014	P > 0,9999
Object	12682	1	12682	F (1, 36) = 88,02	P < 0,0001
**Fig. 4 B**					
ANOVA table	SS	DF	MS	F (DFn, DFd)	P value
Interaction	3,735	1	3,735	F (1, 22) = 0,02683	P = 0,8714
Before/after shock	9491	1	9491	F (1, 22) = 68,17	P < 0,0001
Genotype	1633	1	1633	F (1, 22) = 6,361	P = 0,0194
**Fig. 4 C**					
ANOVA table	SS	DF	MS	F (DFn, DFd)	P value
Interaction	1,466	1	1,466	F (1, 22) = 0,007460	P = 0,9320
Before/after tone	5242	1	5242	F (1, 22) = 26,67	P < 0,0001
Genotype	372,5	1	372,5	F (1, 22) = 2,655	P = 0,1175
**Fig. 4 D**					
ANOVA table	SS	DF	MS	F (DFn, DFd)	P value
Interaction	476,4	1	476,4	F (1, 40) = 3,467	P = 0,0700
Context	8267	1	8267	F (1, 40) = 60,16	P < 0,0001
Genotype	1343	1	1343	F (1, 40) = 9,775	P = 0,0033
**Fig. 5 A**					
ANOVA table	SS	DF	MS	F (DFn, DFd)	P value
Interaction	1,001	1	1,001	F (1, 22) = 0,001922	P = 0,9654
5 min interval	2172	1	2172	F (1, 22) = 4,171	P = 0,0533
Genotype	27,08	1	27,08	F (1, 22) = 0,09763	P = 0,7576
**Fig. 5 B**					
ANOVA table	SS	DF	MS	F (DFn, DFd)	P value
Interaction	69143	1	69143	F (1, 22) = 1,652	P = 0,2121
5 min interval	3827	1	3827	F (1, 22) = 0,09142	P = 0,7652
Genotype	246158	1	246158	F (1, 22) = 3,888	P = 0,0613
**Fig. 5 C**					
ANOVA table	SS	DF	MS	F (DFn, DFd)	P value
Interaction	30,72	1	30,72	F (1, 44) = 0,1831	P = 0,6708

Arm	17128	1	17128	F (1, 44) = 102,1	P < 0,0001
Genotype	24,16	1	24,16	F (1, 44) = 0,1440	P = 0,7062
**Fig. 5 D**					
ANOVA table	SS	DF	MS	F (DFn, DFd)	P value
Interaction	357,1	1	357,1	F (1, 44) = 0,009791	P = 0,9216
Arm	5,916e+006	1	5,916e+006	F (1, 44) = 162,2	P < 0,0001
Genotype	147136	1	147136	F (1, 44) = 4,035	P = 0,0507
**Suppl. Fig. 1 A**					
ANOVA table	SS	DF	MS	F (DFn, DFd)	P value
Interaction	19,80	5	3,960	F (5, 90) = 0,2909	P = 0,9169
Time	64,26	5	12,85	F (5, 90) = 0,9442	P = 0,4565
Genotype	117,1	1	117,1	F (1, 18) = 2,423	P = 0,1370
**Suppl. Fig. 1 B**					
ANOVA table	SS	DF	MS	F (DFn, DFd)	P value
Interaction	19,79	5	3,958	F (5, 90) = 0,2927	P = 0,9159
Time	62,37	5	12,47	F (5, 90) = 0,9226	P = 0,4702
Genotype	122,0	1	122,0	F (1, 18) = 2,610	P = 0,1236
**Suppl. Fig. 1 C**					
ANOVA table	SS	DF	MS	F (DFn, DFd)	P value
Interaction	167,2	5	33,44	F (5, 95) = 1,823	P = 0,1156
Time	479,2	5	95,85	F (5, 95) = 5,226	P = 0,0003
Genotype	145,5	1	145,5	F (1, 19) = 2,396	P = 0,1381
**Suppl. Fig. 1 D**					
ANOVA table	SS	DF	MS	F (DFn, DFd)	P value
Interaction	3005	5	601,1	F (5, 105) = 2,612	P = 0,0287
Time	3676	5	735,1	F (5, 105) = 3,194	P = 0,0100
Genotype	5077	1	5077	F (1, 21) = 7,790	P = 0,0109
**Suppl. Fig. 1 E**					
ANOVA table	SS	DF	MS	F (DFn, DFd)	P value
Interaction	21,87	5	4,374	F (5, 100) = 2,171	P = 0,0633
Time	41,97	5	8,395	F (5, 100) = 4,167	P = 0,0018
Genotype	56,17	1	56,17	F (1, 20) = 9,317	P = 0,0063

SS = sum of squares, DF = degrees of freedom, MS = mean square, DFn = degrees of freedom numerator, DFd = degrees of freedom denominator, P = probability value

Biology Open • Supplementary information

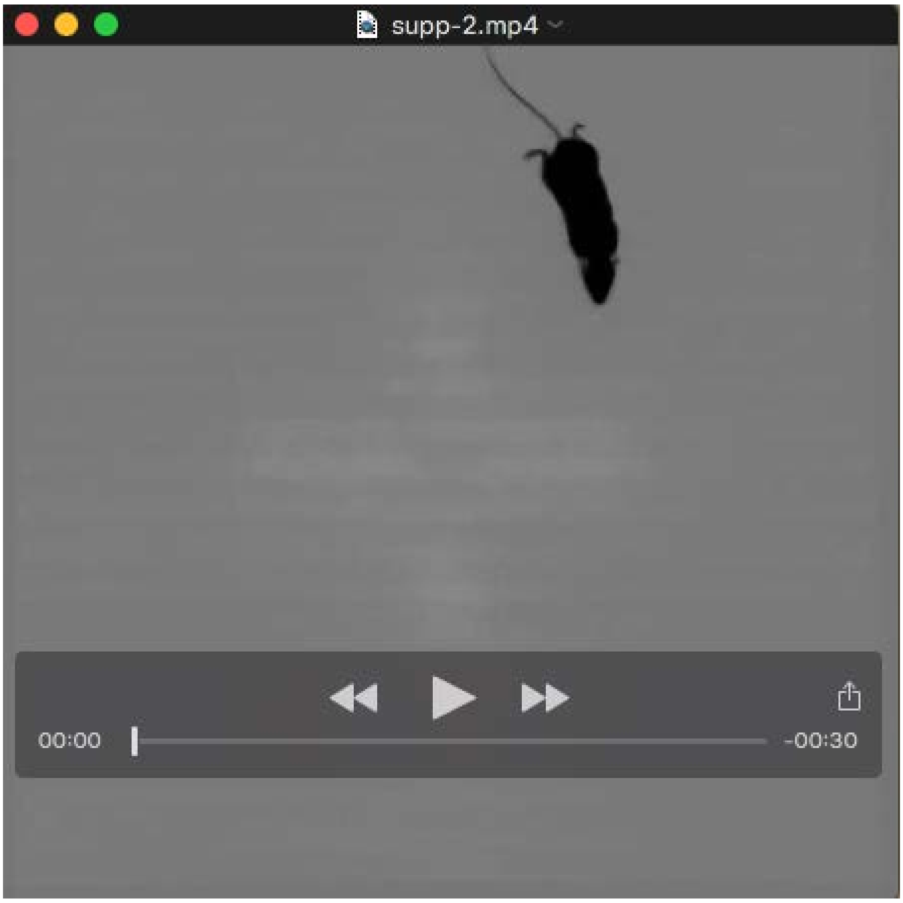

Movie 1: Motor behaviour of *Syap1*$^{Y/-}$ knockout mouse in open field test.

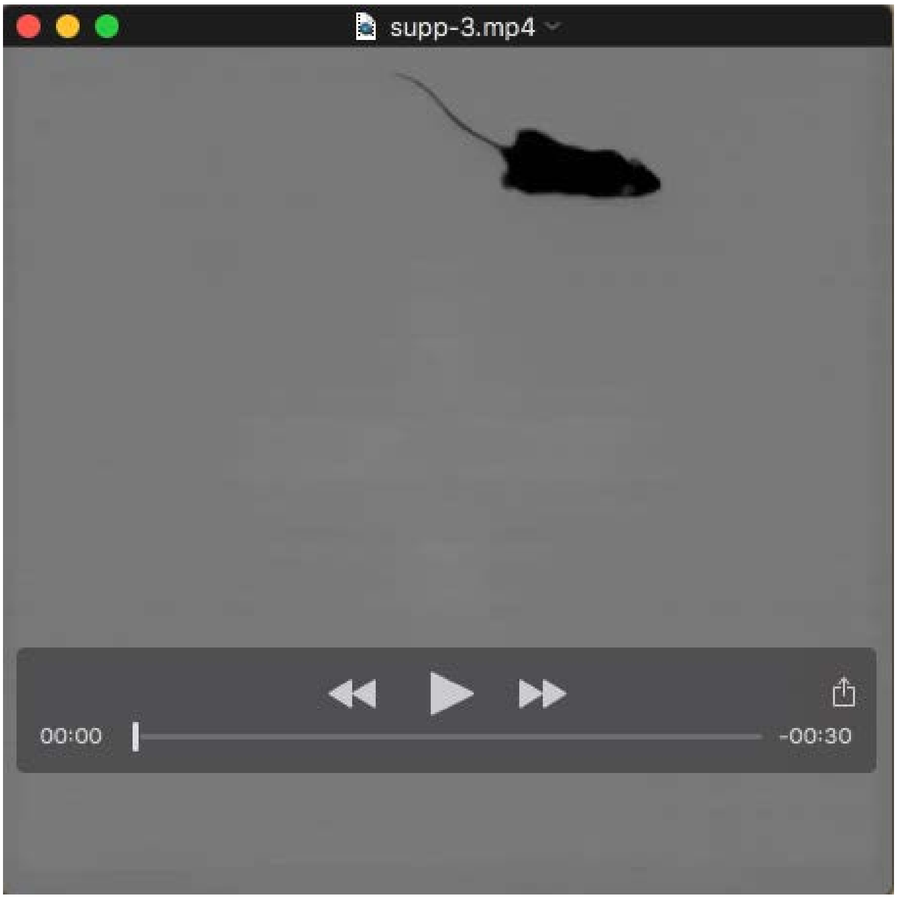

Movie 2: Motor behaviour of wildtype mouse in open field test.
